# Renal Dysfunction and Serum Sodium-Based Risk Stratification for In-Hospital Mortality in Liver Cirrhosis

**DOI:** 10.3390/medicina62071274

**Published:** 2026-07-02

**Authors:** Sonja Golubović, Božidar Dejanović, Dimitrije Damjanov, Nebojša Janjić, Vladimir Veselinov, Gordana Stražmešter-Majstorović, Violeta Knežević

**Affiliations:** 1 Clinic for Nephrology and Clinical Immunology, University Clinical Center of Vojvodina, 21000 Novi Sad, Serbia; vladimir.veselinov@mf.uns.ac.rs (V.V.); gordana.strazmester@kcv.rs (G.S.-M.); violeta.knezevic@mf.uns.ac.rs (V.K.); 2 Faculty of Medicine, University of Novi Sad, 21000 Novi Sad, Serbia; bozidar.dejanovic@mf.uns.ac.rs (B.D.); dimitrije.damjanov@mf.uns.ac.rs (D.D.); nebojsa.janjic@mf.uns.ac.rs (N.J.); 3 Clinic for Gastroenterology and Hepatology, University Clinical Center of Vojvodina, 21000 Novi Sad, Serbia

**Keywords:** liver cirrhosis, renal dysfunction, hyponatremia, MELD, mortality, risk stratification

## Abstract

*Background and Objectives*: Kidney dysfunction and hyponatremia are prevalent in decompensated liver cirrhosis and are associated with suboptimal prognoses. Most prognostic tools used in cirrhosis are hepatic-centric and necessitate the utilization of multiple laboratory and clinical variables. This study endeavored to delineate and assess kidney function- and serum sodium-based prognostic models for the prediction of in-hospital mortality in patients with liver cirrhosis. *Materials and Methods*: This retrospective single-center cohort study comprised 547 hospitalized patients with liver cirrhosis and comprehensive data pertaining to serum urea, creatinine, sodium, age, sex, and in-hospital outcome. In-hospital mortality was the primary endpoint. Kidney function was evaluated via the assessment of serum urea, serum creatinine, and estimated glomerular filtration rate (eGFR), using the Chronic Kidney Disease Epidemiology Collaboration (CKD-EPI) 2021 equation. Simple categorical and point-based scores were constructed. Model discrimination, calibration, and operating characteristics were compared with MELD, Child–Pugh, and ALBI scores, as well as with selected combined models. *Results*: Of the 547 patients, 147 individuals (26.9%) succumbed during the hospitalization period. In the full model, lower eGFR, elevated urea levels, and diminished serum sodium concentrations were independently associated with in-hospital mortality, whereas age and sex did not demonstrate statistical significance. The full model appeared to exhibit moderate discrimination (AUC 0.701, 95% CI 0.652–0.750). A biochemical model based on urea, creatinine and sodium appeared to yield a similar AUC (0.696), and a renal–electrolyte model encompassing eGFR, urea, and sodium seemed to demonstrate an AUC of 0.694. A simple creatinine–sodium score may have attained an AUC of 0.681 and appeared to effectuate the stratification of mortality from 16.4% in the low-risk group to 53.1% in the high-risk group. Adding renal–electrolyte variables or the simple score to MELD did not appear to confer substantial enhancement to performance. *Conclusions*: Kidney dysfunction and hyponatremia at admission have been identified as independent predictors of in-hospital mortality in liver cirrhosis. A simple creatinine–sodium score may afford practical bedside risk stratification and may complement MELD-based assessment in routine clinical care.

## 1. Introduction

Liver cirrhosis represents a significant etiology of morbidity and mortality worldwide [1,2]. Episodes of acute decompensation frequently require hospitalization and are associated with substantial short-term mortality [3,4]. Accurate early risk stratification, therefore, appears essential, as its judicious application may guide monitoring intensity, referral to higher levels of care, and transplant evaluation [5,6].

Prognostic assessment in cirrhosis has traditionally relied upon liver-specific scoring systems [7,8,9,10,11,12]. Prominently, the Child–Pugh score and the Model for End-Stage Liver Disease (MELD) [6] are among the most widely utilized; these instruments serve to summarize hepatic dysfunction and, in the specific context of MELD, integrate renal function via serum creatinine assessment [13]. Furthermore, the albumin–bilirubin (ALBI) score has more recently been employed as an objective hepatic prognostic index [14].

However, patients with decompensated cirrhosis do not succumb solely to hepatic dysfunction [15,16]. Extrahepatic organ dysfunction, particularly renal impairment and electrolyte perturbations, assumes a considerable role [17]. Kidney dysfunction and hyponatremia are among the most clinically relevant abnormalities in advanced cirrhosis [18,19]. Both conditions typically reflect severe circulatory and neurohumoral dysregulation, and their manifestation correlates with the development of ascites, hepatorenal syndrome, hepatic encephalopathy, infection, and, ultimately, mortality.

Within routine clinical practice, particularly across internal medicine and nephrology contexts, early bedside assessment frequently depends upon routine laboratory variables, namely serum urea, creatinine, and sodium, prior to the formal computation of more intricate hepatic scoring systems [20,21]. Simple tools derived from these variables may therefore offer practical value. Nonetheless, the extent to which such kidney-based models may predict short-term mortality, and their comparative efficacy against established hepatic scoring systems, remains to be fully elucidated.

The objective of this investigation involved the development and subsequent evaluation of kidney function- and serum sodium-based prognostic models, including a simple point-based bedside score, for the prediction of in-hospital mortality in patients with liver cirrhosis. Although MELD-Na and MELD 3.0 are available through online calculators, their computation requires bilirubin, INR, and additional variables that may not be immediately available during initial triage. The proposed simple score requires only two routine biochemical values (serum creatinine and sodium), making it potentially applicable in emergency triage or resource-limited settings where comprehensive hepatic scoring is not immediately feasible. A subsequent objective entailed the comparison of their discrimination and calibration with MELD, Child–Pugh and ALBI, as well as with combined models integrating renal–electrolyte variables into MELD.

## 2. Materials and Methods

The present investigation comprised a retrospective, single-center cohort study of consecutive adult patients hospitalized with liver cirrhosis at University Clinical Center of Vojvodina, Clinic for gastroenterology and hepatology in the time period 2020–2025. The analytical framework relied upon a structured clinical and laboratory database, which encompassed demographic characteristics, admission laboratory values, clinical features of decompensation, established liver severity scores and in-hospital outcomes. The study was conducted in accordance with the Declaration of Helsinki and was approved by the Ethics Committee of University Clinical Center of Vojvodina (approval number available upon request). As this was a retrospective review of de-identified, routinely collected clinical data, individual informed consent was waived by the ethics committee in accordance with applicable local regulations.

### 2.1. Study Population

A total of 800 patients with liver cirrhosis hospitalized between 2020 and 2025 were identified from the institutional database and screened for potential inclusion. Of these, 253 were excluded due to missing data on one or more required variables (serum urea, serum creatinine, serum sodium, age, sex, or in-hospital outcome) or because they were formally listed for liver transplantation at the time of admission. The remaining 547 patients satisfied all inclusion criteria and were consequently incorporated into the primary complete-case analysis. A patient selection flowchart is presented in Figure S1. Regarding transplant-related considerations, this study was conducted at a non-transplant hepatology unit. No patients underwent liver transplantation during the index hospitalization, and transplantation was therefore not a competing outcome in this cohort.

### 2.2. Data Collection and Variables

The database encompassed demographic data, the duration of hospitalization, the etiology of cirrhosis, the rationale for admission, comorbidities, routine hematological and biochemical variables, coagulation parameters, clinical manifestations of portal hypertension and decompensation, and in-hospital outcome. Composite liver-related and inflammation-related indices available in the dataset included Child–Pugh class and score, MELD, ALBI, APRI, FIB-4, NLR, LMR and PLR. The assessment of renal function was conducted utilizing serum creatinine, serum urea, and the estimated glomerular filtration rate (eGFR). The eGFR computation was derived from admission serum creatinine through the application of the CKD-EPI 2021 equation, subsequent to the conversion of creatinine from µmol/L to mg/dL.

### 2.3. Outcome Definition

In-hospital mortality constituted the primary outcome measure. For the purpose of regression analyses, death was numerically encoded as one (1) for patients who expired during hospitalization, and as zero (0) for survivors.

### 2.4. Derivation of Simple Score Categories

The derivation of simplified categorical models entailed the categorization of admission serum urea, creatinine, and sodium through the application of predefined clinical thresholds. The urea thresholds (\u22648, 8\u201320, >20 mmol/L) were selected based on published prognostic cut-points in decompensated cirrhosis cohorts. Sex-specific creatinine thresholds were derived from standard laboratory upper limits of normal and the CKD-EPI reference framework. Sodium thresholds corresponded to established clinical definitions of normonatremia (\u2265135 mmol/L), moderate hyponatremia (130\u2013134 mmol/L), and severe hyponatremia (<130 mmol/L) as per published consensus guidance on hyponatremia management in liver disease.

Urea was categorized as:

- 0 points for values ≤8 mmol/L;
- 1 point for values >8 to 20 mmol/L;
- 2 points for values >20 mmol/L.

The categorization of creatinine involved the application of sex-specific thresholds, delineated as follows:

- Men: ≤106 µmol/L = 0 points; 107–150 µmol/L = 1 point; >150 µmol/L = 2 points;
- Women: ≤80 µmol/L = 0 points; 81–120 µmol/L = 1 point; >120 µmol/L = 2 points.

The classification of sodium levels proceeded as follows:

- 0 points were assigned for values ≥135 mmol/L;
- 1 point for values 130–134 mmol/L;
- 2 points for values <130 mmol/L.

The evaluation encompassed two simplified scoring systems, specifically:

- Simple score 2 = creatinine category + sodium category (range 0–4);
- Simple score 3 = urea category + creatinine category + sodium category (range 0–6).

For purposes of clinical interpretation, the categorization of simple score 2 delineated risk into three strata: low risk (zero to one), intermediate risk (two to three), and high risk (four); similarly, simple score 3 was classified into low risk (zero to one), intermediate risk (two to three), and high risk (four or greater).

### 2.5. Statistical Analysis

Continuous variables underwent summarization through medians with interquartile ranges, while categorical variables were presented as counts and percentages; moreover, a descriptive comparison was conducted between survivors and non-survivors.

The analysis involved the fitting of several logistic regression models, employing in-hospital death as the dependent variable, delineated as follows:

- Full model: estimated Glomerular Filtration Rate (eGFR), urea, sodium, age, sex;
- Biochemical model: urea, creatinine, sodium;
- Renal–electrolyte model: estimated Glomerular Filtration Rate (eGFR), urea, sodium;
- Simple categorical model: urea category, creatinine category, sodium category;
- Simple score 2 and simple score 3 as ordinal predictors.

The reporting of odds ratios, accompanied by their respective 95% confidence intervals, was undertaken. Quantification of discrimination was achieved utilizing the area under the receiver operating characteristic curve (AUC) and its associated 95% confidence intervals. AUC values were derived from logistic regression-predicted probabilities for each regression model; for established scores (MELD, Child–Pugh, ALBI), AUC was computed by treating the score value directly as the predictor in an ROC analysis. Confidence intervals for AUC were obtained using the DeLong method, and pairwise AUC comparisons between models were performed using the DeLong test for correlated ROC curves. Calibration was assessed using the Brier score and the Hosmer–Lemeshow goodness-of-fit test. The identification of optimal clinical cut-offs was performed employing the Youden index. Furthermore, the calculation of sensitivity, specificity, positive predictive value, negative predictive value, and accuracy was also conducted.

The performance of kidney-based models underwent comparison with the Model for End-Stage Liver Disease (MELD), Child–Pugh, and Albumin–Bilirubin (ALBI) scores. Specifically, combined models encompassed MELD and Child–Pugh, MELD and ALBI, MELD and renal–electrolyte variables, and MELD and simple score three. A *p* value < 0.05 was considered statistically significant.

## 3. Results

### 3.1. Patient Characteristics

A total of 547 patients with liver cirrhosis were included in the complete-case analysis. Among them, 147 patients (26.9%) died during hospitalization. The median age demonstrated similarity between survivors and non-survivors. The distribution of sexes, moreover, exhibited no statistically significant divergence (Table 1).

Non-survivors manifested a more pronounced renal dysfunction and an exacerbated electrolyte imbalance upon admission. The median urea concentration was 18.7 mmol/L in non-survivors, compared with 8.9 mmol/L in survivors. Median creatinine levels were 146 µmol/L versus 92 µmol/L. Median sodium concentrations were 131 mmol/L versus 136 mmol/L. The median eGFR was 41 mL/min/1.73 m^2^ versus 72 mL/min/1.73 m^2^. Liver-specific scores also exhibited a deterioration in patients who succumbed, characterized by elevated MELD and Child–Pugh values and diminished ALBI values.

### 3.2. Full Renal–Electrolyte Model and Related Models

Within the comprehensive model, which incorporated the estimated glomerular filtration rate (eGFR), urea, sodium, age, and sex, lower eGFR, elevated urea, and diminished sodium levels were independently associated with in-hospital mortality (Table 2). Age and sex did not demonstrate statistical significance.

The full model seemingly exhibited moderate discrimination, evidenced by an AUC of 0.701 (95% CI 0.652–0.750). Calibration appeared satisfactory, as indicated by a Brier score of 0.173 and a non-significant Hosmer–Lemeshow test.

A biochemical model, which incorporated urea, creatinine, and sodium, produced a comparable AUC of 0.696. A renal–electrolyte model, encompassing eGFR, urea, and sodium, demonstrated an AUC of 0.694.

### 3.3. Simple Categorical Models and Point-Based Scores

In the categorical model based on categorized urea, creatinine, and sodium, the creatinine and sodium categories demonstrated an independent association with mortality, whilst the urea category did not achieve statistical significance. This model achieved an AUC of 0.688. Simple score 3 attained an AUC of 0.682, whereas simple score 2 yielded an AUC of 0.681; nevertheless, despite their inherent simplicity, these point-based models retained a substantial proportion of the prognostic signal observed within the comprehensive model.

**Figure 1:**
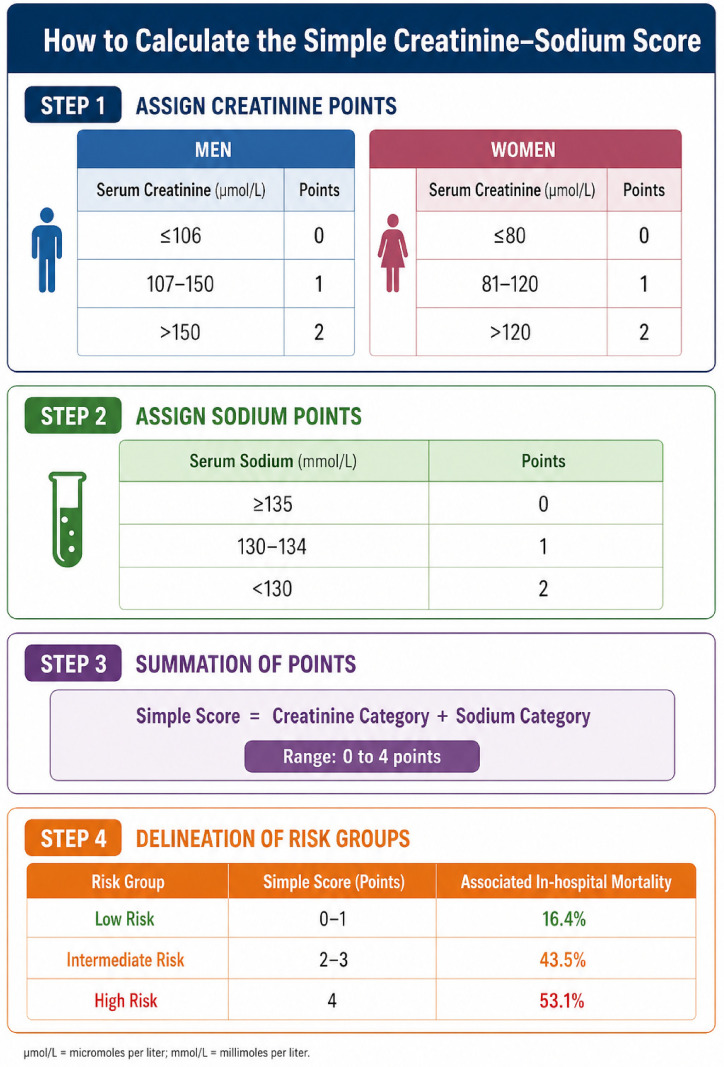
How to calculate a simple score model.

For simple score 2, mortality increased progressively across score values, with score 0: 15.7%, score 1: 18.0%, score 2: 42.1%, score 3: 46.8%, score 4: 53.1%. When grouped into clinically defined strata, simple score 2 showed low risk (0–1): 16.4% mortality; intermediate risk (2–3): 43.5% mortality; high risk (4): 53.1% mortality. All models are represented in Table 3, as well as Figure 1.

### 3.4. Comparison with Established Liver Scores

**Figure 2:**
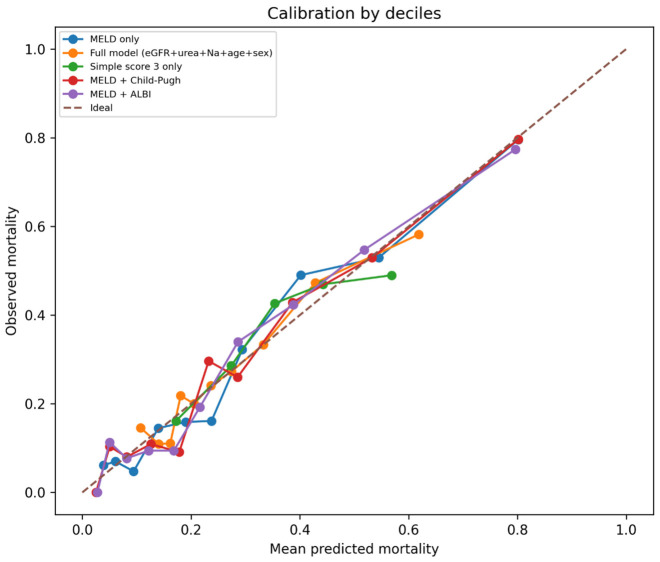
Calibration of models.

**Figure 3:**
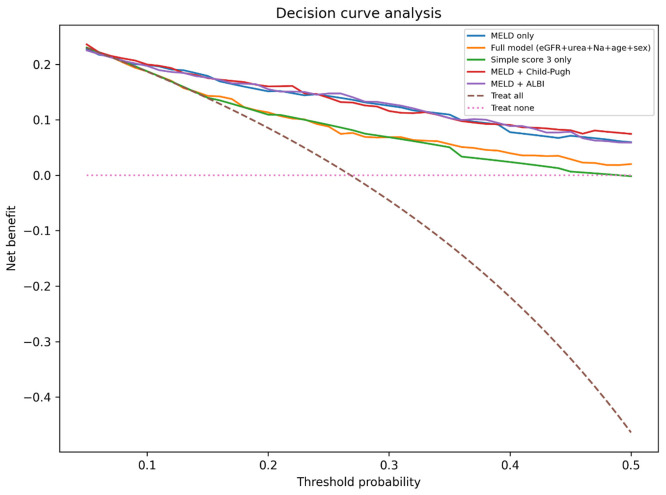
Comparison of laboratory/based models and scores.

Among individual liver-related scores, Model for End-Stage Liver Disease (MELD) demonstrated the highest discriminatory capacity for in-hospital mortality, with an AUC of 0.814. Child–Pugh score yielded an AUC of 0.760, and the Albumin–Bilirubin (ALBI) index an AUC of 0.714. The kidney-based and simple models had AUCs between approximately 0.68 and 0.70. These results are illustrated in Figure 2. A simplified comparison of laboratory-based models and scores is shown in Figure 3.

### 3.5. Incremental Value Beyond MELD

**Figure 4:**
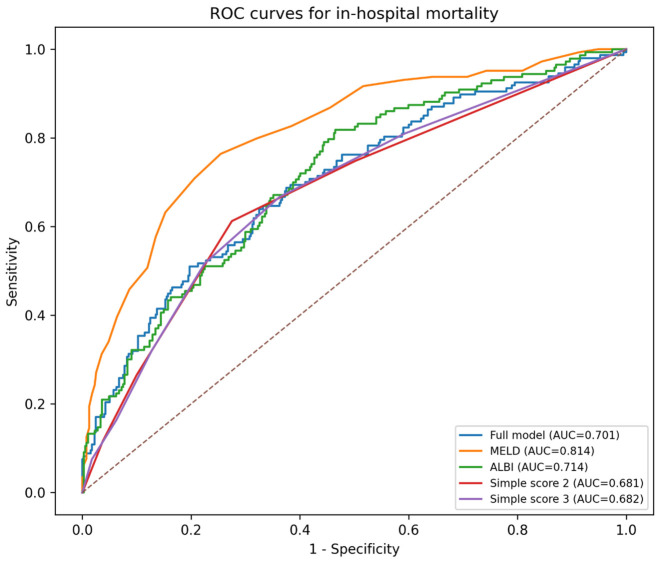
Decision curve analysis.

An evaluation of combined models was undertaken to ascertain whether the inclusion of renal–electrolyte variables or simple scores could enhance prediction beyond that afforded by MELD. MELD + Child–Pugh had the highest AUC (0.825), followed closely by MELD + ALBI (0.824). In contrast, MELD + renal–electrolyte variables and MELD + simple score 3 had AUCs that were essentially identical to MELD alone. These findings might suggest that, whilst renal dysfunction and hyponatremia appear to be clinically relevant predictors of mortality, they may not materially improve discrimination once MELD’s impact is accounted for. Decision curve analysis demonstrating the clinical utility of the evaluated models is presented in Figure 4.

## 4. Discussion

### 4.1. Principal Findings

The present investigation assessed kidney function- and sodium-based prognostic models in hospitalized patients with liver cirrhosis. The primary findings suggest several key observations: first, a reduced estimated Glomerular Filtration Rate (eGFR), an elevated urea concentration, and diminished serum sodium levels appeared to be independently associated with in-hospital mortality; second, renal–electrolyte models demonstrated moderate discriminative capacity; third, a simplified creatinine–sodium score may facilitate clinically meaningful bedside stratification; and fourth, the Model for End-Stage Liver Disease (MELD) score largely maintained its preeminence as an optimal individual prognostic index.

The observed in-hospital mortality of 26.9% appears largely consistent with reported mortality rates in hospitalized cohorts with acute decompensation [22]. Our MELD AUC of 0.814 is also in line with published short-term prognostic studies, where MELD often performs in the 0.75–0.90 range depending on disease severity and study endpoint [23]. This observation, therefore, lends support to the plausibility of the present findings and may underscore the considerable prognostic utility of MELD in advanced cirrhosis. While MELD demonstrates robust prognostic capabilities, the European Association for the Study of the Liver-CLIF consortium’s Acute-on-Chronic Liver Failure classification appears to demonstrate superior accuracy for predicting 28-day and 90-day mortality, particularly when patients are stratified by ACLF classification at 48 h post-enrollment [24].

### 4.2. The Contextualization of Renal Dysfunction

The independent association between renal dysfunction and mortality in this cohort is consistent with the established conceptualization that the kidney is a major determinant of outcome in cirrhosis, alongside hepatic parameters such as serum albumin which similarly reflect disease severity and prognosis [17]. Renal injury in cirrhosis reflects complex circulatory and inflammatory disturbances and is closely linked with hepatorenal syndrome, infection and multiorgan failure [25]. Prior studies have shown that acute kidney dysfunction markedly worsens short-term prognosis [24]. The present findings extend this observation through the demonstration that even baseline admission indices, including estimated glomerular filtration rate (eGFR) and urea, may convey independent prognostic information regarding in-hospital mortality. Furthermore, the predictive utility of renal markers may be enhanced through the incorporation of novel biomarkers like cystatin C, which has demonstrated improved prognostic accuracy when substituted for creatinine in the MELD score [26]. Conversely, the CLIF-C ACLF score and the CLIF-C AD score appear to exhibit significantly greater accuracy than MELD and MELD-Na scores in predicting short-term and long-term mortality among patients with and without acute-on-chronic liver failure (ACLF), respectively [27]. Collectively, these scores, alongside the CLIF-C Organ Failure score, demonstrably exhibit superior performance in predicting mortality among cirrhotic patients, thereby outperforming other established scoring systems, such as MELD, MELD-Na, and Child–Pugh, particularly within contexts of acute-on-chronic liver failure [28].

The performance of our renal–electrolyte models, with AUC values around 0.69–0.70, appears comparable to the range reported for numerous non-MELD prognostic tools in decompensated cirrhosis [13]. This observation thus suggests that the prognostic contribution of renal dysfunction, as observed herein, is not anomalous but rather constitutes an integral component of a broader, reproducible pattern consistently documented across hospital-based investigations.

Hyponatremia in cirrhosis is indicative of advanced circulatory dysfunction and compromised free water excretion [29]. Its association with ascites, hepatic encephalopathy, hepatorenal syndrome, and increased mortality has been established [29]; furthermore, prior investigations have demonstrated that each decrement in serum sodium concentration correlates with an unfavorable outcome, a correlation that ultimately precipitated the development of MELD-Na [29,30]. The present findings thus reiterate the clinical significance of hyponatremia as an independent prognosticator of adverse outcomes among cirrhotic patients, thereby reinforcing the utility of sodium-inclusive scoring systems [31].

In the present study, sodium appeared independently associated with in-hospital death within the comprehensive model, additionally demonstrating substantial contributive capacity to the simple bedside score. This observation may underscore the clinical relevance of sodium not only as a marker of disease severity but also as a practical component in the stratification of short-term risk. Nevertheless, the predictive advantage of serum sodium may diminish in patients presenting with higher MELD scores, suggesting its utility in individuals with less severe liver disease [32]. This observation appears consistent with findings in other cohorts wherein the incremental prognostic value of sodium, extending beyond MELD, is most pronounced among patients exhibiting lower MELD scores, thereby substantially contributing to a more refined risk assessment within this particular subgroup [33]. The incorporation of serum sodium levels into predictive models, such as the MELD-Na score, has been posited to enhance the prediction of short-term mortality in patients with liver disease; evidence from alcoholic hepatitis cohorts supports that serum sodium adds meaningful prognostic information beyond MELD alone [34].

### 4.3. Comparison with MELD, Child–Pugh and ALBI

MELD emerged as the strongest single prognostic score within this cohort; a finding that may not elicit surprise. MELD incorporates creatinine, thereby potentially encompassing a substantial portion of the risk associated with renal dysfunction. The absence of relevant improvement when renal–electrolyte variables or the simple score were added to MELD likely reflects this fact. Indeed, the MELD score’s objective and continuous variables make it a robust predictor of mortality in liver cirrhosis. Furthermore, serum sodium level has been independently associated with the severity of cirrhosis-related complications, underscoring its prognostic significance beyond MELD alone. An important limitation of the present analysis is that MELD-Na and MELD 3.0 were not formally calculated and compared as separate models. MELD-Na, which incorporates serum sodium, and MELD 3.0, which adds further clinical and sex-related parameters, represent the current recommended standards for prognostic assessment in cirrhosis. Direct comparison with these more contemporary MELD variants would have strengthened the conclusions, and their omission is acknowledged as a methodological limitation. Nevertheless, given that the sodium variable is already incorporated into MELD-Na, it is expected that the simple creatinine–sodium score would not outperform MELD-Na. Its intended role is as a rapid, first-contact triage instrument in settings where formal MELD calculation is not immediately feasible [35]. The integration of serum sodium into the MELD score to form MELD-Na further enhances prognostic accuracy, particularly in predicting waiting list mortality for liver transplantation [36,37].

Child–Pugh and ALBI demonstrated an intermediate performance profile. Their AUC values, as observed within this cohort, appear compatible with the broad range delineated in preceding studies [38]. Importantly, the simple creatinine–sodium score performed only modestly worse than these established liver scores, despite relying on only two routine laboratory variables and no clinical grading. This aspect may represent the primary practical contribution of the present investigation [39].

The score, therefore, is not purported to supersede MELD, Child–Pugh, or ALBI; rather, its utility may reside in its capacity as a rapid, minimal-data bedside instrument. This particular applicability may manifest as especially advantageous beyond specialized hepatology settings, specifically where expedited triage is necessitated and comprehensive liver-oriented scoring mechanisms remain inaccessible.

### 4.4. Clinical Implications

The findings possess substantial practical implications. Early assessment of renal dysfunction and hyponatremia is warranted for all hospitalized patients presenting with cirrhosis. Patients with hyponatremia may represent a high-risk subgroup [38]. The simple creatinine–sodium score may facilitate the expedited identification of such patients.

The score’s utility may be particularly pronounced in emergency departments, general medical wards and non-transplant hospitals. The score necessitates solely the utilization of basic admission laboratory data. It is capable of identifying patients, within its higher categories, who exhibit an anticipated mortality rate exceeding 40–50%. This may support earlier escalation of monitoring, closer hemodynamic assessment, avoidance of nephrotoxins, and timely nephrology consultation.

### 4.5. Limitations

Several limitations characterize the present study. Its design was retrospective and single-center, relying upon complete-case analysis; consequently, residual confounding and limited generalizability cannot be definitively excluded. The study’s focus was exclusively on admission values and in-hospital mortality; it did not encompass the evaluation of dynamic renal trajectories, acute kidney injury progression, or longer-term outcomes. Finally, creatinine-based assessment of renal function may be inherently imperfect in cirrhosis, particularly in individuals with diminished muscle mass. A further important limitation is the absence of MELD-Na and MELD 3.0 calculation and direct comparison in the primary analysis, as these represent current standards of care that incorporate sodium and additional variables. The simple creatinine–sodium score proposed herein should therefore be understood as a complementary bedside tool rather than a replacement for these validated models. Additionally, since MELD already incorporates creatinine as a component, the incremental prognostic value of further renal parameters over MELD is inherently constrained, which may limit the novelty of renal–electrolyte models as standalone prognostic tools. Prospective, multicenter validation and direct comparison with MELD-Na and MELD 3.0 are necessary before clinical implementation of the proposed score.

## 5. Conclusions

Among hospitalized patients presenting with hepatic cirrhosis, the presence of renal dysfunction and hyponatremia upon admission appears to constitute independent predictors of in-hospital mortality. The Model for End-Stage Liver Disease (MELD) score, however, remains the strongest solitary prognostic indicator. Nevertheless, a simple creatinine–sodium score does provide practical and clinically meaningful bedside risk stratification; its application may complement MELD-based assessment within routine clinical care.

## Figures and Tables

**Table 1:** Baseline Clinical and Laboratory Characteristics of Survivors and Non-Survivors.

Variable	Survivors (n = 400)	Non-Survivors (n = 147)	*p*-Value
Age (years), mean ± SD	61.8 ± 10.9	63.1 ± 11.2	0.214
Male sex, n (%)	268 (67.0%)	102 (69.4%)	0.598
Urea (mmol/L), median (IQR)	8.9 (6.2–14.5)	18.7 (12.4–29.8)	<0.001
Creatinine (µmol/L), median (IQR)	92 (71–121)	146 (104–210)	<0.001
Sodium (mmol/L), median (IQR)	136 (133–139)	131 (128–134)	<0.001
eGFR (mL/min/1.73 m^2^), median (IQR)	72 (52–91)	41 (26–63)	<0.001
MELD score, median (IQR)	16 (12–21)	24 (19–31)	<0.001
ALBI score, mean ± SD	−1.68 ± 0.52	−1.21 ± 0.61	<0.001

**Table 2:** Discriminative Performance of Liver-Specific and Kidney-Based Models for In-Hospital Mortality.

Variable	OR	95% CI	*p*-Value
eGFR (per mL/min)	0.982	0.974–0.991	<0.001
Urea (per mmol/L)	1.043	1.028–1.058	<0.001
Sodium (per mmol/L)	0.942	0.913–0.971	<0.001
Age (per year)	1.012	0.994–1.031	0.182
Male sex	1.11	0.74–1.67	0.613

**Table 3:** In-hospital mortality by simple creatinine–sodium score categories.

Model	AUC (95% CI)
Full model	0.701 (0.661–0.741)
Categorical model	0.680 (0.642–0.718)
Simple score 2	0.681 (0.643–0.719)
Simple score 3	0.682 (0.644–0.720)
MELD	0.814 (0.780–0.848)
ALBI	0.708 (0.671–0.745)
MELD + Child–Pugh	0.825
MELD + ALBI	0.824

## Data Availability

The data presented in this study are available on request from the corresponding author.

## Supplementary Materials

The following supporting information can be downloaded at: https://www.mdpi.com/article/10.3390/medicina62071274/s1, Figure S1. Patient selection flowchart (STROBE-compliant).

## Abbreviations

The following abbreviations are used in this manuscript:

ALBI	Albumin–Bilirubin
APRI	Aspartate Aminotransferase to Platelet Ratio Index
AUC	Area Under the Curve
CI	Confidence Interval
CKD-EPI	Chronic Kidney Disease Epidemiology Collaboration
eGFR	Estimated Glomerular Filtration Rate
FIB-4	Fibrosis-4 Index
IQR	Interquartile Range
LMR	Lymphocyte-to-Monocyte Ratio
MELD	Model for End-Stage Liver Disease
NLR	Neutrophil-to-Lymphocyte Ratio
NPV	Negative Predictive Value
OR	Odds Ratio
PLR	Platelet-to-Lymphocyte Ratio
PPV	Positive Predictive Value
ROC	Receiver Operating Characteristic
SD	Standard Deviation
ACLF	Acute-on-Chronic Liver Failure
CLIF-C	Chronic Liver Failure Consortium score
CLIF-C AD	CLIF Consortium Acute Decompensation score
