# Supplementary material for: Renal Dysfunction and Serum Sodium-Based Risk Stratification for In-Hospital Mortality in Liver Cirrhosis
Source: Medicina (Kaunas). 2026 Jul 2;62(7):1274. doi: 10.3390/medicina62071274 (PMC13413595; doi:10.3390/medicina62071274)
Supplement: Supplementary file 1 [file medicina-62-01274-s001.zip › medicina-4322082-supplementary.pdf]

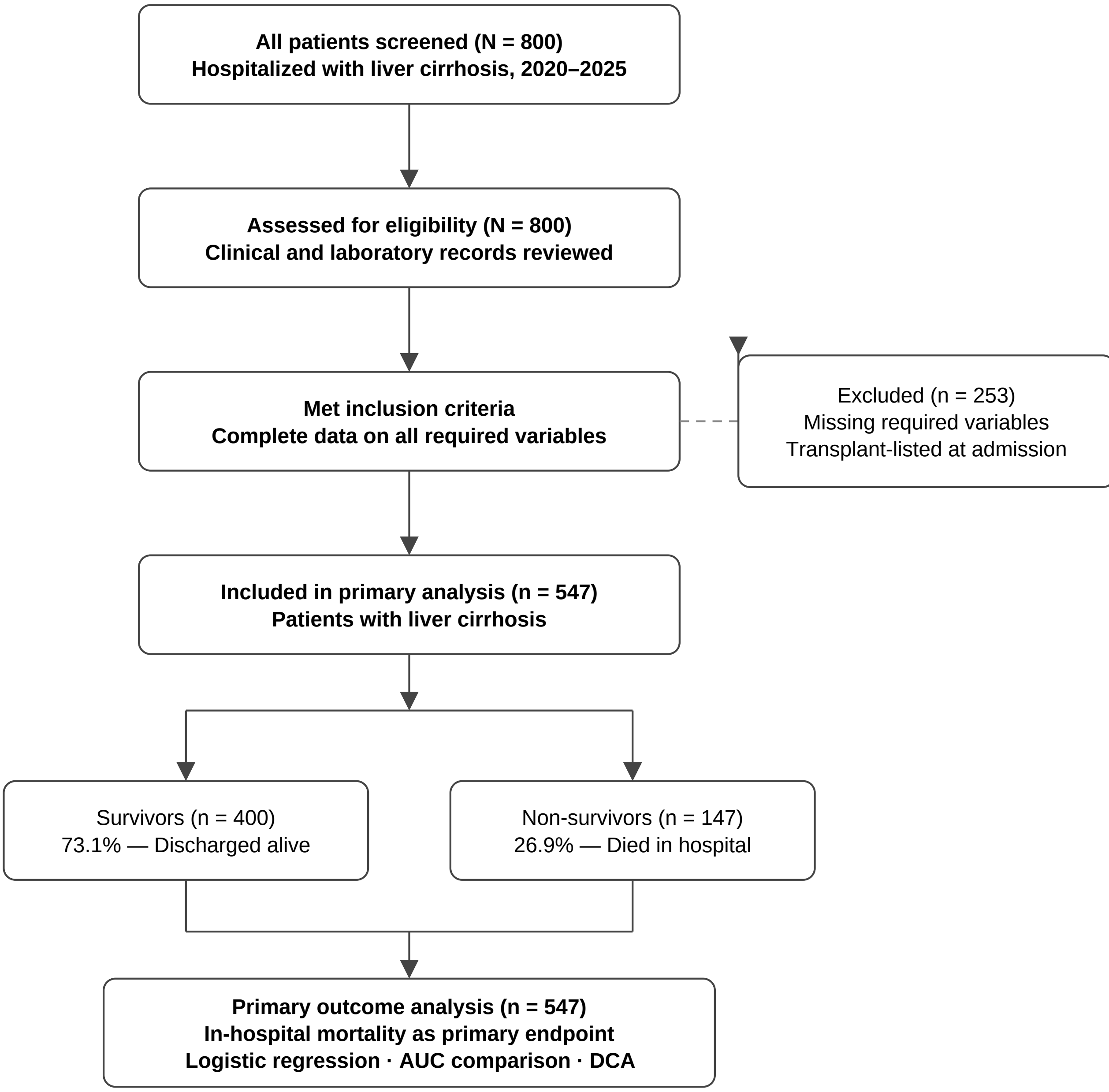

† No patients underwent liver transplantation during the index hospitalization (non-transplant center).

Figure S1. Patient selection flowchart (STROBE-compliant). Medicina 2026.
